# How radical is radical cure? Site-specific biases in clinical trials underestimate the effect of radical cure on *Plasmodium vivax* hypnozoites

**DOI:** 10.1186/s12936-021-04017-1

**Published:** 2021-12-20

**Authors:** John H. Huber, Cristian Koepfli, Guido España, Narimane Nekkab, Michael T. White, T. Alex Perkins

**Affiliations:** 1 Department of Biological Sciences and Eck Institute for Global Health, University of Notre Dame, Notre Dame, IN USA; 2 Unité Malaria: Parasites et Hôtes, Département Parasites et Insectes Vecteur, Institut Pasteur, Paris, France

**Keywords:** *Plasmodium vivax*, Radical cure, Clinical trials, Bias

## Abstract

**Background:**

*Plasmodium vivax* blood-stage relapses originating from re-activating hypnozoites are a major barrier for control and elimination of this disease. Radical cure is a form of therapy capable of addressing this problem. Recent clinical trials of radical cure have yielded efficacy estimates ranging from 65 to 94%, with substantial variation across trial sites.

**Methods:**

An analysis of simulated trial data using a transmission model was performed to demonstrate that variation in efficacy estimates across trial sites can arise from differences in the conditions under which trials are conducted.

**Results:**

The analysis revealed that differences in transmission intensity, heterogeneous exposure and relapse rate can yield efficacy estimates ranging as widely as 12–78%, despite simulating trial data under the uniform assumption that treatment had a 75% chance of clearing hypnozoites. A longer duration of prophylaxis leads to a greater measured efficacy, particularly at higher transmission intensities, making the comparison between the protection of different radical cure treatment regimens against relapse more challenging. Simulations show that vector control and parasite genotyping offer two potential means to yield more standardized efficacy estimates that better reflect prevention of relapse.

**Conclusions:**

Site-specific biases are likely to contribute to variation in efficacy estimates both within and across clinical trials. Future clinical trials can reduce site-specific biases by conducting trials in low-transmission settings where re-infections from mosquito bite are less common, by preventing re-infections using vector control measures, or by identifying and excluding likely re-infections that occur during follow-up, by using parasite genotyping methods.

**Supplementary Information:**

The online version contains supplementary material available at 10.1186/s12936-021-04017-1.

## Background

*Plasmodium vivax* is the most geographically widespread cause of human malaria, and its burden in 2017 was estimated at 14.3 million clinical cases globally [1]. Control of vivax malaria is challenging due to a unique life stage of the parasite, known as the hypnozoite [2], which latently infects the liver of individuals with recent *P. vivax* blood-stage infections [3]. Hypnozoites activate to cause successive relapsing infections following the initial blood-stage infection, and relapses are thought to comprise an estimated 79–96% of all *P. vivax* infections [4, 5]. The prevention of relapses is therefore an ongoing priority for vivax malaria control [6], and clearance of hypnozoites can be achieved through radical cure treatment with an 8-aminoquinoline, such as primaquine (PQ) or tafenoquine (TFQ).

Recent clinical trials for PQ and TFQ have been conducted in Latin America, sub-Saharan Africa and Southeast Asia [7–10]. The DETECTIVE trial estimated that the recurrence-free efficacies of PQ and TFQ were 74 and 70%, respectively [6], suggesting a potential large impact of radical cure as a first-line vivax malaria treatment [11]. However, each trial noted substantial geographical variation in efficacy estimates. Although potentially reflective of intrinsic differences in hypnozoite clearance with 8-aminoquinoline treatment among the distinct trial populations [12] or distinct parasite strains [13], the geographical variation in efficacy may have instead been attributable to features of each transmission setting, which would bias the efficacy estimates and limit the interpretability of trial results when evaluating the extent to which radical cure prevents relapse.

**Fig. 1:**
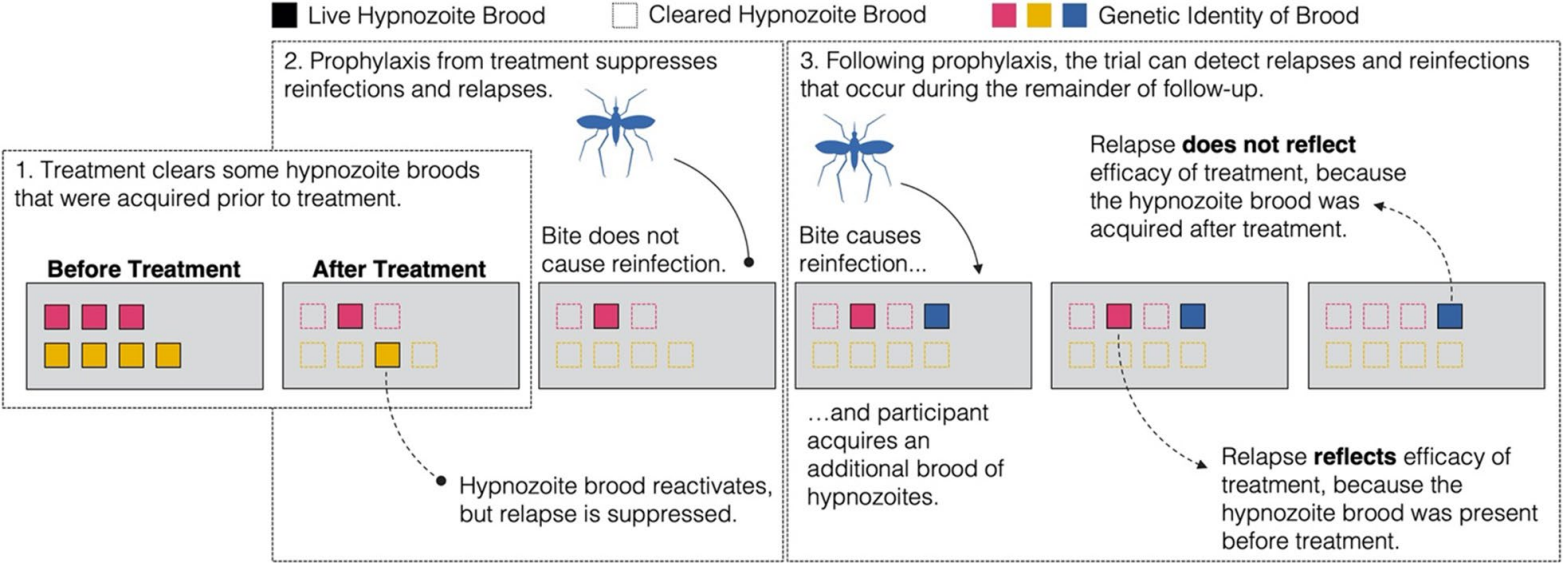
Schematic of potential infection outcomes during trial follow-up. Participants are enrolled in the trial and initially harbour hypnozoite broods (coloured squares). The colours represent the genetic identity of each hypnozoite brood. Hypnozoite broods are cleared (unfilled dotted squares) with 8-aminoquinoline treatment according to the per-hypnozoite brood probability of clearance. Treatment with an 8-aminoquinoline provides each participant with a period of prophylaxis during which reinfections (solid line) and relapses (dotted line) are suppressed. Following prophylaxis, trial participants can experience re-infections and relapses, and these re-infections and relapses may be detected during the remainder of follow-up. Only relapses that arise from the activation of hypnozoite broods that were present before 8-aminoquinoline treatment reflect the per-hypnozoite brood clearance probability

The primary endpoint used in recent clinical trials is freedom from *P. vivax* recurrent infection [7–9], which encompasses relapses initiated by hypnozoite activation, re-infections caused by mosquito biting, and recrudescences caused by blood-stage therapeutic failure [14]. Only relapses arising from hypnozoite broods (i.e., a group of hypnozoites that derives from one infectious mosquito bite) present prior to treatment directly reflect the extent to which 8-aminoquinoline treatment prevents relapse, or the hypnozoite brood clearance probability (hereafter, clearance probability) (Fig. 1). Because there remains no reliable way to distinguish these relapses from all other recurrent infections using epidemiological data alone, efficacy against recurrent infection is not equal to efficacy against relapse, and the measured efficacy will likely vary depending upon the site where the trial is conducted. Obtaining standardized estimates of the effect of each 8-aminoquinoline against hypnozoite broods will therefore depend upon the proportion of recurrent infections identified during follow-up that are relapses associated with hypnozoite broods acquired prior to 8-aminoquinoline treatment.

An incongruence between the primary endpoint and the effect that the clinical trial intends to measure has previously been noted as a source of bias in determining the therapeutic cure rates for falciparum malaria and tuberculosis [15–17]. The magnitude of this bias could depend upon features of the trial location and the trial design. Although most or all clinical vivax malaria patients enrolled in clinical trials are expected to carry hypnozoites, the rates at which participants experience re-infections and relapses may vary across trial sites due to various epidemiological features, including transmission intensity, heterogeneous mosquito biting patterns [18], and the relapse phenotype of the *P. vivax* parasite [19]. The number of recorded relapses also depends upon the duration of follow-up and the duration of prophylaxis provided by the treatment regimen used in the trial. The extent to which these different features of the trial location and the trial design contribute to bias in efficacy estimates, and impact the ability to measure the clearance probability, has not been explored.

Here, an existing and validated individual-based model of *P. vivax* transmission [11, 20] is extended to simulate clinical trials for radical cure. Individual-based models have been used to design and evaluate clinical trials, both for malaria [21, 22] and other infectious diseases [23–26]. To maintain consistency with previous trials, the clinical trials were modelled on the recent DETECTIVE trials for PQ and TFQ [7–9]. By varying different features of the trial location and trial design, this analysis sought to understand how site-specific biases in efficacy estimates may arise. It also explored different approaches, such as the use of vector control and parasite genotyping methods, that could be implemented to mitigate site-specific biases and standardize estimates of the effect of radical cure against hypnozoite broods.

## Methods

### Transmission model

To simulate *P. vivax* transmission, this study used a stochastic, individual-based model developed by White et al. [11] and Nekkab et al. [20]. This model extends the Ross-MacDonald framework of *Plasmodium falciparum* transmission to incorporate relapses [27], a characteristic feature of *P. vivax* transmission [28]. Calibrated to epidemiological surveys conducted in Papua New Guinea, the Solomon Islands and Brazil, the model by White et al*.* [11] and Nekkab et al. [20] reproduces *P. vivax* transmission dynamics across a range of epidemiological settings.

The rates at which individuals in the population are re-infected and relapse depend upon the respective number of infectious mosquito bites received (i.e., the entomological inoculation rate, (EIR)) and the number of hypnozoite broods present in each individual’s liver. The average EIR within the population is determined by the mosquito population dynamics, and individual variation therein is due to heterogeneity in exposure and age-dependent differences in biting. Following successful inoculation with *P. vivax* sporozoites from an infectious mosquito bite, an individual accumulates an additional brood of hypnozoites within the liver and experiences a primary blood stream infection. In the absence of treatment, each brood of hypnozoites can activate to cause relapses or be cleared naturally from the liver at fixed rates (Additional file 1: Table S1).

The nature of each blood-stage infection (i.e., re-infection or relapse) is determined by the level of anti-parasite and clinical immunity in the infected individual. In the model, the level of anti-parasite immunity determines the probability that a blood-stage infection is detectable by light microscopy (LM) and increases the rate at which low-density infections are cleared. The level of clinical immunity determines the probability that an individual with a blood-stage infection exhibits symptoms.

The levels of anti-parasite and clinical immunity interact to determine the detectability of each blood-stage *P. vivax* infection. The model propounded by White et al*.* [11] and Nekkab et al. [20] considers three types of blood-stage infections: (1) sub-microscopic infections detectable by PCR; (2) sub-clinical infections detectable by LM and PCR; and, (3) clinical infections detectable by LM and PCR. Following White et al. [11] and Nekkab et al. [20], it was assumed that each clinical infection was characterized by a fever exceeding 38 °C within the past 48 h and a parasite density greater than 500/µL. Individuals with clinical *P. vivax* infections then seek treatment with anti-malarial drugs according to a probability of treatment-seeking behaviour.

Simulation of the *P. vivax* transmission model occurs in two steps. First, the population is initialized at equilibrium according to the analogous set of deterministic compartmental differential equations. Then, the stochastic, individual-based model is simulated with the initialized population. For further description of the model and its assumptions, please refer to the Supplement and the documentation provided in White et al*.* [11] and Nekkab et al. [20].

### Trial overview

This analysis constructed clinical trials for a generic 8-aminoquinoline (e.g., PQ or TFQ). Trials were simulated in order to quantify the recurrence-free efficacy as a measure of the therapeutic effect of radical cure on the hypnozoite broods present in each individual presenting with a clinical *P. vivax* infection confirmed by light microscopy.

The simulated clinical trials were designed to be comparable to previous trials of PQ and TFQ [7–9]. Participants were randomly assigned to the treatment and control arms, and all participants received chloroquine (CQ) upon enrolment to clear the *P. vivax* blood-stage parasites. Consistent with the DETECTIVE trial [7, 9], participants were followed for 180 days and assessed for asexual parasitaemia by light microscopy at days 8, 15, 22, 29, 60, 90, 120, and 180 post-enrolment. For trials simulated with longer duration of follow-up, additional time points were included. See the Supplement for further details.

### Radical cure model

The analysis modelled the action of radical cure in each individual receiving treatment as the per-hypnozoite brood probability of clearance. To allow for heterogeneous action of the intervention [29], it was assumed that a proportion ${p}_{i}$ of the population belonged to each stratum *i* with per-hypnozoite brood clearance probability, ${c}_{i}$. For a population consisting of two strata, the mean per-hypnozoite brood probability of clearance was

1 $$\bar{c}={{p}_{1}}{{c}_{1}}+{{p}_{2}}{{c}_{2}},$$

where ${p}_{2}=1-{p}_{1}$. This model of heterogeneous action of radical cure generalized the intervention actions commonly considered in the evaluation of clinical trials. Under the ‘leaky’ model [29–31], the action of radical cure is the same across all individuals in the population (${p}_{1}=1$) with $0\le {c}_{1}\le 1$. Under the ‘all-or-none’ model [29–31], radical cure completely clears hypnozoites in a sub-set of the population ($0<{p}_{1}<1;{c}_{1}=1$) and has no effect on the hypnozoites in the remainder of the population (${p}_{2}=1-{p}_{1};{c}_{2}=0$). These two models of intervention action could manifest due to host-specific factors, most notably the cytochrome P-450 isoenzyme 2D6 (CYP2D6) genotype, an enzyme involved in metabolizing PQ. For individuals with a low CYP2D6 metabolization phenotype, evidence suggests that PQ may not prevent *P. vivax* blood-stage relapses [12], though its precise effect on 8-aminoquinoline efficacy remains poorly understood. For example, under an all-or-none action, CYP2D6 metabolization could manifest as a binary phenotype with high metabolizers effectively clearing hypnozoite broods and low metabolizers failing to clear hypnozoite broods. Alternatively, under a leaky action, all treated individuals partially clear hypnozoite broods. Supplementary analyses revealed that the choice of intervention action did not substantially affect the efficacy estimates generated using the Cox proportional hazards model (Additional file 1: Fig. S5). The minimal differences in efficacy between the all-or-none and leaky intervention actions are attributable to the recurrent infection endpoint (Additional file 1: Fig. S9), which includes re-infections by mosquito biting that are not affected by the choice of intervention action. When excluding re-infections, differences were captured in the survival curves between the two intervention actions (Additional file 1: Fig. S10). Because this analysis used a recurrent infection endpoint, it therefore assumed by default that each 8-aminoquinoline had an all-or-none action with ${p}_{1}=0.75$ and ${c}_{1}=1$.

Under the model of heterogeneous action of radical cure, the number of broods of hypnozoites following treatment was distributed as

2 $${H}_{post} \sim Binomial\left({H}_{pre},1-{c}_{i}\right),\forall i\in \left\{\mathrm{1,2}\right\}.$$

In Eq. (2), ${H}_{pre}$ is the number of broods of hypnozoites present in the liver prior to treatment, and *c*_*i*_ is the per-hypnozoite brood clearance probability for an individual in stratum *i*.

### Trial design

The simulated clinical trials for radical cure were constructed to match the trial design used in past trials for PQ and TFQ [7–9]. Accordingly, the simulations considered four phases of the randomized control trial design: (1) recruitment; (2) treatment; (3) vector control; and, (4) follow-up.

### Recruitment

Individuals were enrolled in the simulated clinical trial if they presented at a health clinic with febrile illness and were diagnosed by light microscopy with a *P. vivax* infection. Consistent with the DETECTIVE trial [7, 9], participants were enrolled if they were at least 16 years of age and had a measured glucose-6-phosphate dehydrogenase (G6PD) activity greater than 70% of the median value of the trial location. Following Nekkab et al. [20], it was assumed that G6PD activity was measured using the SD Biosensor STANDARD G6PD test [32]. This test qualitatively classifies G6PD activity as normal (> 70% activity), intermediate (30–70% activity), and low (< 30% activity).

Individuals that met the criteria for enrolment were then randomly assigned to the treatment or control arm of the clinical trial. Allocation of participants to each trial arm occurred with equal probability, provided that the current number of participants in each arm was less than the desired sample size of 1000.

### Treatment

Upon enrolment, all trial participants were treated with a 3-day course of CQ to clear the blood-stage, asexual parasites [7, 9]. This analysis assumed that treatment with CQ was 100% effective and that there were no recrudescences among trial participants. Participants in the treatment arm were also treated with an 8-aminoquinoline to clear the hypnozoite reservoir. For individuals in the treatment arm, the number of hypnozoite broods following radical cure was calculated according to Eq. (2). It was further assumed that treatment with the 8-aminoquinoline provided both blood-stage and liver-stage prophylaxis. Consistent with White et al*.* [11] and Nekkab et al. [20], the duration of prophylaxis was 28 days for PQ when co-administered with CQ and 45 days for TFQ when co-administered with CQ.

### Vector control

As in the DETECTIVE trial [7, 9], all participants were provided with a long-lasting insecticidal net (LLIN) to prevent re-infection from mosquitoes during follow-up and therefore decrease the potential bias that may arise in calculating efficacy based on a recurrent infection endpoint. Following Griffin et al. [33] and White et al. [11], it was assumed that vector control measures decrease the probabilities of mosquito biting and successful feeding and increase the probabilities of repellency and death. The magnitude of the effect depends upon the proportion of mosquito bites that occur while individuals are indoors (${\Phi }_{I}$) and in bed (${\Phi }_{B}$), the duration of usage of the LLIN, and the duration of insecticidal activity. The probability of usage decayed exponentially over time with a half-life of 3 years. The simulated clinical trials further assumed that insecticidal activity decayed exponentially over time with a half-life of 2.5 years [20].

This analysis also considered whether the use of indoor residual spraying (IRS) in each participant’s house administered in isolation or in combination with LLINs decreased the potential bias in efficacy estimates. As with LLINs, it was assumed that the use of IRS decreases the probabilities of mosquito biting and successful feeding and increases the probabilities of repellency and death [11, 33]. To account for the waning effect of IRS over time, insecticidal activity decayed exponentially with a half-life of 6 months [20].

### Follow-up

Each participant was followed for 180 days following enrolment in the clinical trial and treatment with anti-malarial drugs. Consistent with the DETECTIVE trial [7, 9], for each participant, simulations recorded the date of each clinical *P. vivax* infection episode that occurred within the duration of follow-up. Furthermore, each participant was tested for *P. vivax* asexual parasites using light microscopy on days 8, 15, 22, 29, 60, 90, 120, 160, and 180 post-enrolment. No mortality among trial participants was assumed during the follow-up period.

To examine potential biases that arise from trial surveillance methods, the simulated clinical trials kept a complete record of all recurrent infections that occurred within the duration of follow-up, including those that would not have been detected under the trial protocol. For each recurrent infection, the cause (i.e., re-infection or relapse) and the type of blood-stage infection (i.e., sub-microscopic, sub-clinical or clinical) was recorded. The simulations further distinguished between relapses associated with hypnozoite broods acquired prior to treatment and relapses associated with hypnozoite broods acquired following treatment. Only relapses associated with hypnozoite broods acquired prior to treatment reflect the action of 8-aminoquinoline treatment against hypnozoite broods.

### Statistical analyses

This analysis calculated the efficacy of the 8-aminoquinoline used in each clinical trial from the output collected in each respective simulation. It calculated efficacy using multiple metrics in order to examine how different data collected during trial follow-up resolved biases in the efficacy estimates.

Consistent with previous clinical trials [7, 9], freedom from LM-detectable recurrent infection was used as the default efficacy metric in the simulation studies. Efficacy was calculated using the Cox proportional hazards model [34], which computes the hazard ratio between the treatment and control arms based on the times to first LM-detectable recurrent infection. Following the DETECTIVE trial protocol [7, 9], recurrent infections that occurred before 32 days post-enrolment were not included. This left-censoring period was applied by the trial investigators to exclude recrudescences associated with blood-stage therapeutic failure, though these infection types were not simulated in the model. Given that the timing of each recurrent infection was unobserved, all trial participants were interval censored.

This analysis calculated efficacy using incidence rates computed from the recurrent infections that occurred within the duration of follow-up. Efficacy was calculated based on incidence rates as

3 $${E}_{IR}=1-{\frac{{{e}_{t}}/{{y}_{t}}}{{{e}_{c}}/{{y}_{c}}}}$$

In Eq. (3), ${e}_{t}$ and ${e}_{c}$ are the respective number of infection events in the treatment and control arms, and ${y}_{t}$ and ${y}_{c}$ are the respective number of person-years of follow-up in the treatment and control arms [31, 34].

The magnitude of the measured efficacy also depends upon the action of the radical cure therapeutic. In the absence of other sources of bias, efficacy will be overestimated for 8-aminoquinolines that elicit an all-or-none response if efficacy is calculated based on incidence rates. This phenomenon is caused by the sub-set of individuals in the treatment arm who completely clear the hypnozoites from their liver and increase the number of person-years of follow-up [31]. To resolve this bias that arises from the assumption of intervention action, this analysis calculated efficacy based on risk as

4 $${E}_{R}=1-\frac{{p}_{t}}{{p}_{c}},$$

where ${p}_{t}$ and ${p}_{c}$ are the proportion of individuals in the treatment and control arms that experience a particular infection event within the duration of follow-up.

### Simulation experiments

This analysis performed simulation experiments to quantify biases in the efficacy estimates that may arise under different transmission settings and other features of the clinical trial design (Table 1). First, it considered how the efficacy estimates varied with transmission intensity and heterogeneity in mosquito biting patterns. Second, it considered how differences in the rate of relapse of the *P. vivax* hypnozoites interact with the duration of the trial follow-up and the transmission intensity to bias estimates of efficacy. After characterizing these biases, it assessed whether the allocation of vector control measures, such as LLINs and IRS, to trial participants could prevent re-infections and thereby reduce the bias in the efficacy estimates. This analysis next considered whether a method using time-to-event and genotyping data that are capable of distinguishing between different types of *P. vivax* recurrent infections at different sensitivities and specificities could reduce or correct the bias in the efficacy estimates. Then, it measured the extent to which the duration of prophylaxis provided by the treatment regimen biases estimates of efficacy by preventing re-infections in the treatment arm only. Finally, it considered how the efficacy estimates that was obtained varied with the choice of efficacy metric and infection endpoint. A supplementary analysis exploring a sub-set of these effects was also performed using a simpler, hazard-based transmission model.

**Table 1:** Parameters varied during the simulation analyses

Variance in mosquito biting	Time to relapse (d)	Follow-up (d)	Prophylaxis (d)
Effect of transmission intensity and heterogeneity in biting			
0, 1, 2, 3	65	180	28
Effect of rate of relapse and duration of follow-up			
0	30, 60, 90, 180	90, 180, 365, 730	28
Effect of vector control/genotyping/efficacy metric and infection endpoint			
0	65	180	28
Effect of the 8-aminoquinoline			
0	65	180	28, 45

The parameters that were varied during each simulation analysis are reported. All other parameters in the transmission model are set to default values and are consistent with the values reported in Additional file 1: Tables S1 and S2

For each simulation setting, a clinical trial with 1000 participants in each arm was simulated. This sample size was chosen to ensure that the power of the clinical trials exceeded 95% for each transmission intensity and efficacy metric considered (Additional file 1: Fig. S6). Each population was simulated for 30 years prior to trial enrolment to ensure that transmission stabilized. Each trial was simulated in a population of 200,000 individuals, and the maximum duration of each trial was 5 years in order to ensure that complete enrolment was attained, even at low transmission intensities. By default, PQ was the 8-aminoquinoline provided to the participants in the treatment arm and when co-administered with CQ provided 28 days of blood-stage and liver-stage prophylaxis during which trial participants could not become re-infected or relapse. The mean per-hypnozoite brood probability of clearance was equal to 0.75. For each simulation setting, trials were simulated in which the therapeutic had an all-or-none response. Because the transmission model was stochastic, 200 simulations were run for each simulation setting, and the mean and interquartile range for the efficacy estimates across these simulations was computed. By default, this analysis defined efficacy as freedom from LM-detectable recurrent infection calculated using the Cox proportional hazards model.

### Availability of code

All code to reproduce the analyses in this study is available on GitHub at https://github.com/johnhhuber/Radical_Cure_Uncertainty.

## Results

### Effect of transmission intensity and heterogeneity in biting

This analysis first examined how transmission intensity and the level of heterogeneity in mosquito biting patterns in the trial location interact to affect efficacy estimates. Transmission intensity was varied by setting the EIR to 1, 10 or 100 infectious bites per person-year, and the level of heterogeneity in mosquito biting was varied by changing the variance in the distribution of individual-level exposure to mosquito biting.

**Fig. 2:**
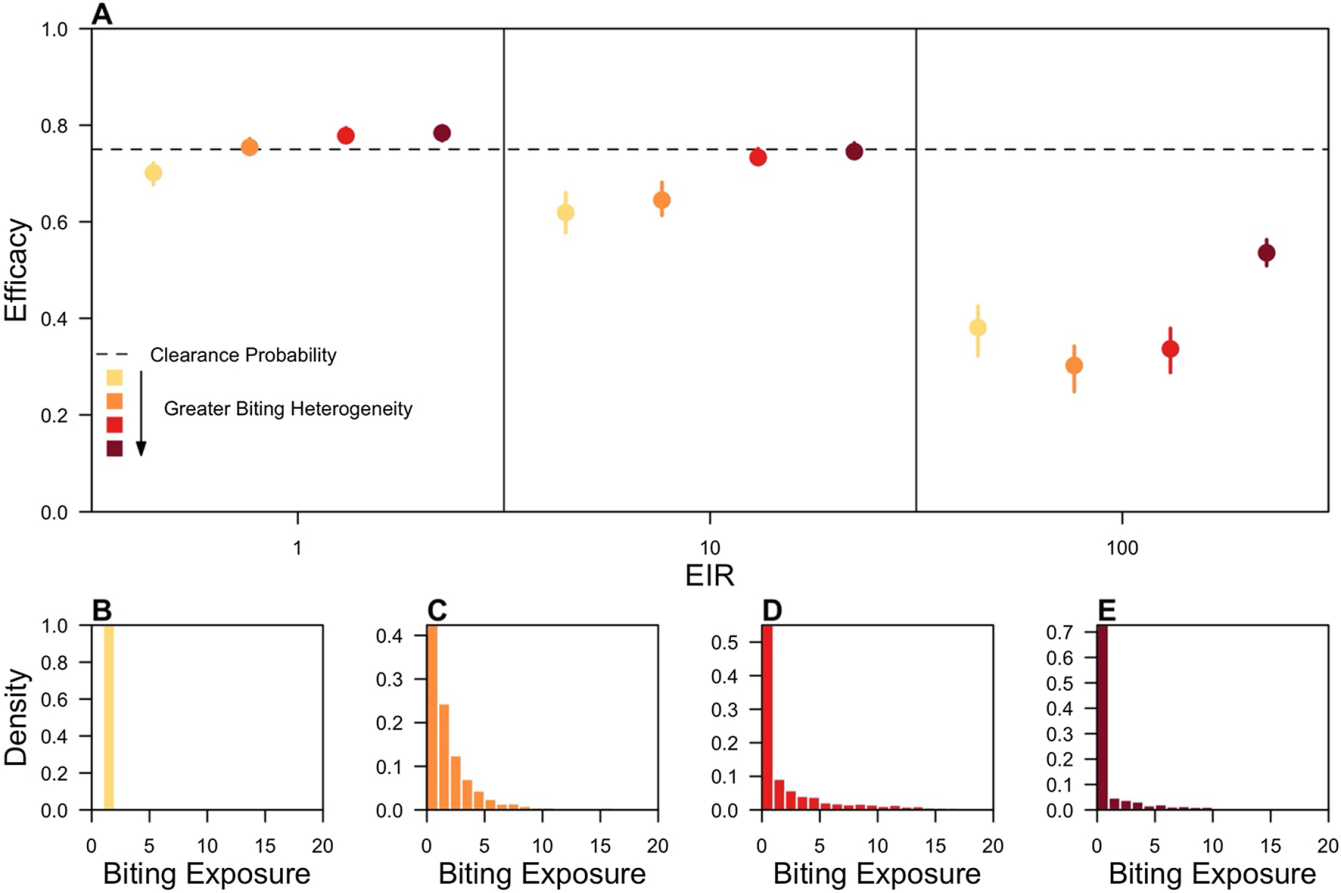
Effect of transmission intensity and heterogeneity in biting on efficacy estimates. **A** LM-detectable recurrence-free efficacy estimated from simulated clinical trials is shown at different EIR and levels of heterogeneity in biting. Each point represents the median of 200 simulations, and each bar is the interquartile range. The colour represents the degree of heterogeneity in individual-level exposure to biting, corresponding to the distributions in **B**–**E**. Darker colours indicate greater heterogeneity in individual-level exposure to biting, and the dotted line is the clearance probability. The distributions of biting propensities are shown from representative simulated trials where the variance in the logged biting propensity was equal to (**B**) zero, (**C**) one, (**D**) two, or (**E**) three

**Fig. 3:**
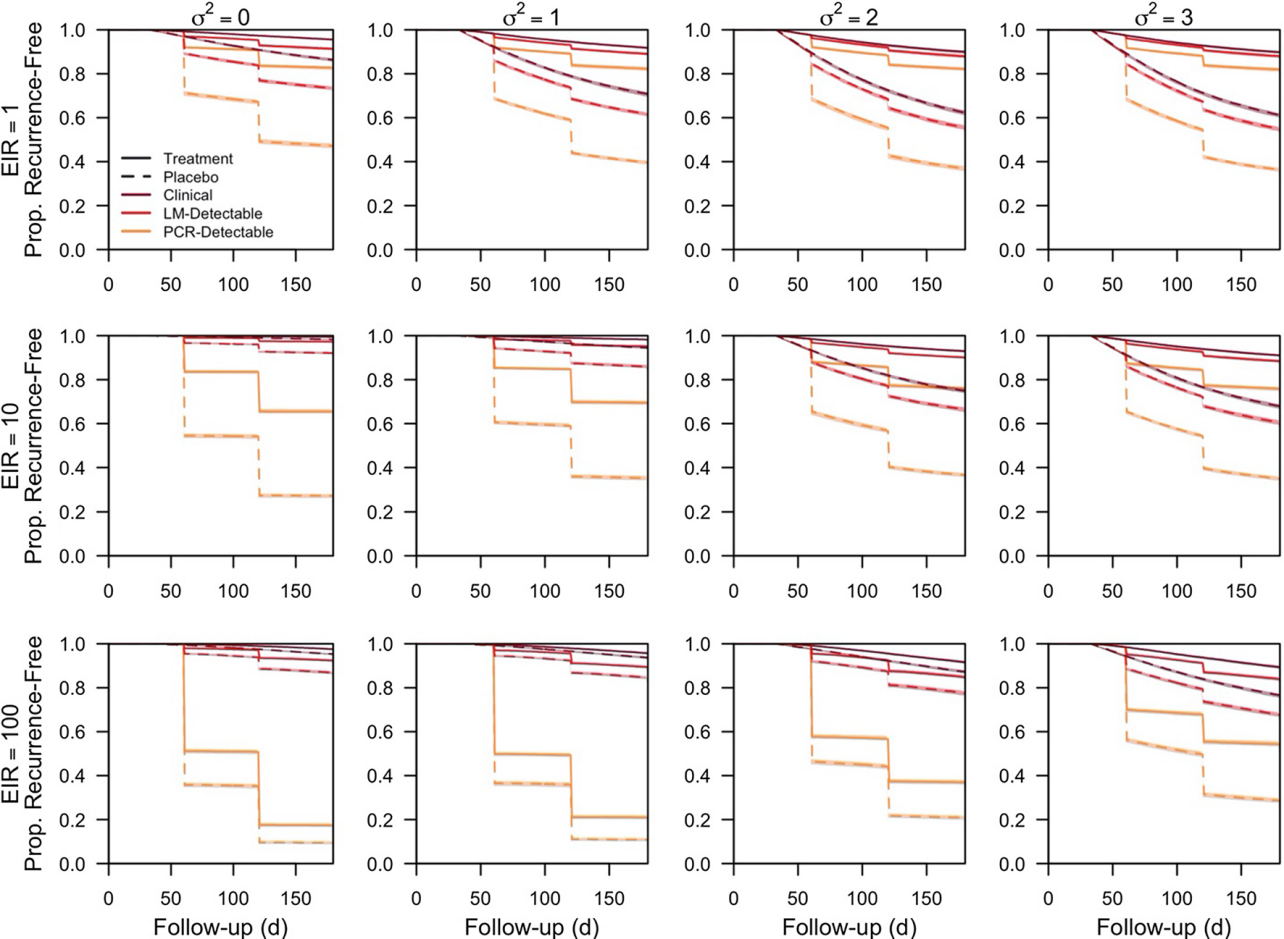
Recurrence-free survival curves for the effect of transmission intensity and heterogeneity in biting. Recurrence-free survival from simulated clinical trials as a function of follow-up time is shown at different EIR and levels of heterogeneity in biting (σ^2^). Each line is in the median of 200 simulations, and each shaded region is the interquartile range. Solid lines correspond to the treatment arm, and dashed lines correspond to the placebo arm. The colour of each survival curve corresponds to the infection endpoint used, with orange corresponding to PCR-detectable recurrent infections, red corresponding to LM-detectable recurrent infections, and maroon corresponding to clinical recurrent infections

When exposure to mosquito biting was the same for all trial participants (Fig. 2B), a downward bias in efficacy estimates was observed that increased with transmission intensity (Fig. 2A). For clinical trials of an 8-aminoquinoline with a clearance probability equal to 0.75, LM-detectable recurrence-free efficacies ranged from 0.70 (IQR: 0.68–0.72) at an EIR of 1 to 0.38 (0.32–0.43) at an EIR of 100. This downward bias in efficacy occurred due to both frequent re-infection, an infection event that does not reflect the clearance probability, and the reduced detectability of recurrent infections due to higher levels of anti-parasite immunity at higher transmission intensities. As transmission intensity increases, more trial participants were re-infected by mosquitoes, yet parasite densities were reduced and fewer recurrent infections were LM-detectable (Fig. 3, left column), causing the infection profiles of the treatment and control arms to appear more similar and leading to a lower measured efficacy.

At low transmission intensities when re-infection rates were low and heterogeneity in biting was high, efficacy estimates exceeded the clearance probability, revealing a positive bias. This positive bias occurred because it was assumed that radical cure completely cleared hypnozoite broods in a sub-set of trial participants and did not clear hypnozoite broods in the remainder of trial participants. In trials measuring time to first recurrent infection, those participants that completely clear hypnozoite broods increase the total person-time at risk in the treatment arm, reducing the estimated hazard of recurrent infection in the treatment arm and leading to a competing positive bias that was present in all trial simulations. The magnitude of this bias increases with follow-up time, provided that participants are not re-infected by mosquito biting, and was eliminated if efficacy was instead calculated based upon the proportion at risk using the complete record of recurrent infections (Additional file 1: Fig. S1).

In addition to the effect of transmission intensity itself, the effect of heterogeneous biting on efficacy estimates was modulated by the transmission intensity of the trial location. At EIRs of 1 and 10, the downward bias was reduced with greater heterogeneity in biting. Efficacy measured at an EIR of 10 ranged from 0.62 (0.58–0.66) under homogeneous biting to 0.75 (0.73–0.76) under the highest level of heterogeneity in biting simulated. Heterogeneous biting reduced the downward bias associated with frequent reinfection, because fewer re-infections from mosquito biting occurred during follow-up. As exposure decreased on average with greater heterogeneity in biting, anti-parasite immunity levels were lower, and a greater proportion of recurrent infections were LM-detectable (Fig. 3). The improved detectability of recurrent infections revealed the differences in the infection profiles across the trial arms, reducing the downward bias due to transmission intensity. At an EIR of 100, the effect of heterogeneous biting on efficacy estimates was non-monotonic (Fig. 2A). Although the percentage of participants re-infected during follow-up decreased monotonically with greater heterogeneity in mosquito biting, the percentage of participants with a detectable re-infection (i.e., clinical or sub-clinical/LM-detectable) varied non-monotonically and was 36% (35–36%), 39% (38–40%), 35% (35–36%), and 25% (24–25%) when the respective variance in exposure to biting was 0, 1, 2, and 3 (Fig. 2B–E).

The effects of transmission intensity and heterogeneity in biting on efficacy estimates were also observed using a simple, hazard-based model (Additional file 1: Fig. S12). That these downward biases could be captured using a model that ignores many complexities of *P. vivax* transmission further corroborates these observations.

### Effect of the rate of relapse and duration of follow-up

This analysis next examined how the rate of relapse of *P. vivax* hypnozoites and the duration of follow-up of trial participants interact to affect efficacy estimates. Mean times to relapse of 30, 60, 90, and 180 days were considered, and clinical trials were simulated in which participants were followed for 90, 180, 365, or 730 days. To assess the effect of transmission intensity, all trials were simulated at EIRs of 1, 10, and 100, assuming homogeneous biting.

**Fig. 4:**
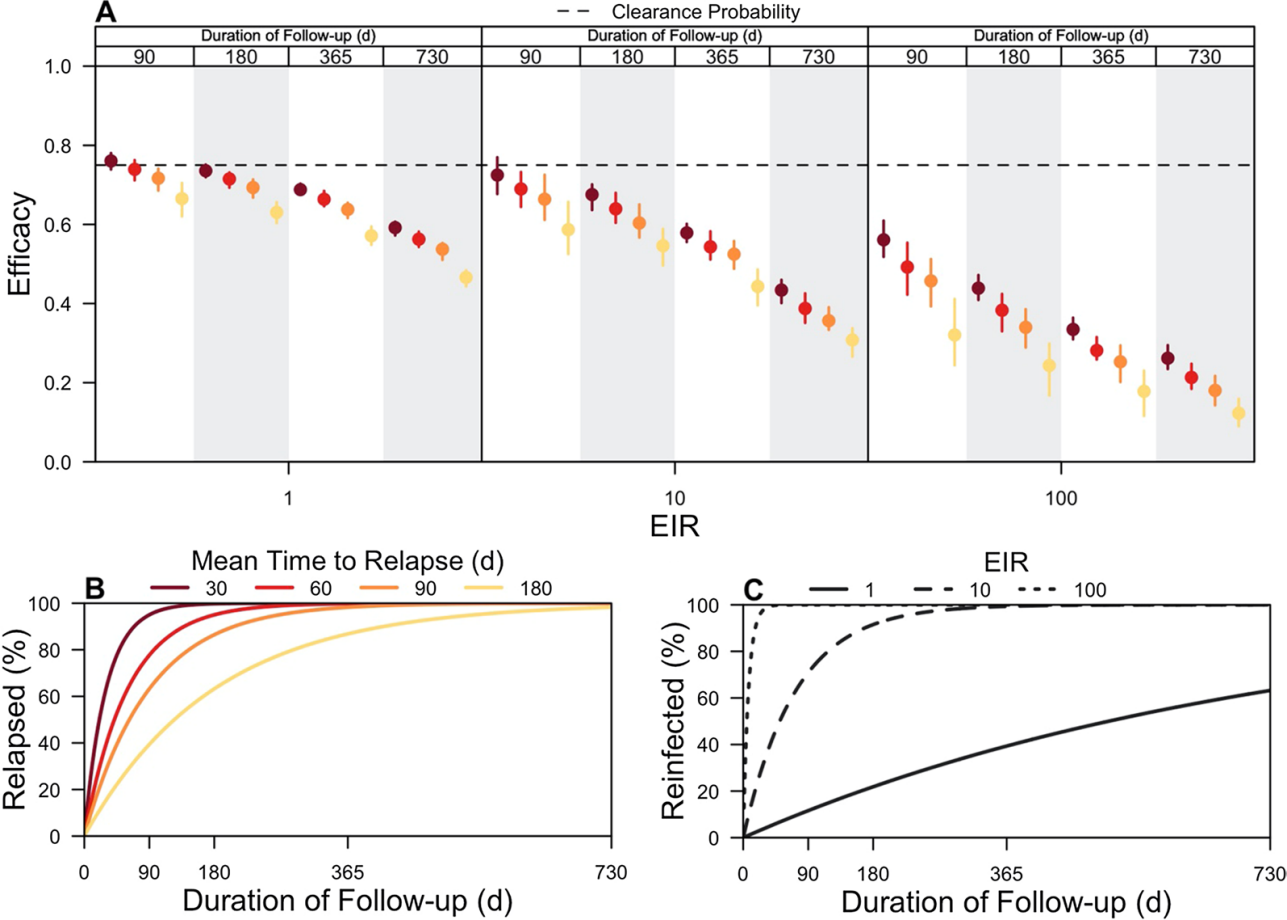
Effect of rate of relapse and duration of follow-up on efficacy estimates. **A** LM-detectable recurrence-free efficacy estimated from simulated clinical trials is shown at different EIRs, mean times to relapse, and durations of follow-up. Each point is the median of 200 simulations, and each bar is the interquartile range. The color represents the mean time to relapse (30, 60, 90, or 180 days), and each vertical strip for each EIR is the duration of follow-up (90, 180, 365, or 730 days). The dotted line is the clearance probability simulated in each trial. **B** The percentage of trial participants expected to relapse as a function of the duration of follow-up is shown for different mean times to relapse. **C** The percentage of trial participants expected to be re-infected as a function of the duration of follow-up is shown for different EIRs

For a given duration of follow-up, efficacy estimates decreased with a longer mean time to first relapse (Fig. 4A). In simulated trials in which participants were followed for 180 days at an EIR of 1, the estimated efficacy of an 8-aminoquinoline with a clearance probability of 0.75 decreased from 0.74 (IQR: 0.72–0.75) to 0.63 (0.60–0.66) as the mean time to first relapse increased from 30 to 180 days. This downward bias occurred because, as the mean time to first relapse increased, fewer trial participants were expected to relapse within the duration of follow-up (Fig. 4B), causing the distribution of times to first observed recurrent infection to appear more similar across trial arms (Additional file 1: Fig. S2, top row). Only 63.2% of participants were expected to relapse by 180 days when the mean time to first relapse was 180 days, compared to 99.8% of participants when the time to first relapse was 30 days.

For a given mean time to first relapse, efficacy estimates decreased with a longer duration of follow-up (Fig. 4A). With an EIR equal to 1 and a mean time to first relapse of 30 days, the estimated efficacy of an 8-aminoquinoline was 0.76 (0.74–0.78) when trial participants were followed for 90 days, compared to 0.59 (0.57–0.61) when trial participants were followed for 730 days. Under an alternative scenario in which the EIR was 10, the estimated efficacies were 0.72 (0.68–0.77) and 0.43 (0.40–0.46) at the respective durations of follow-up. The downward bias occurred because a longer duration of follow-up ensured that more trial participants were re-infected by mosquito biting (Fig. 4C), similarly causing the distribution of times to first observed recurrent infection to appear more similar across the trial arms. By 90 days of follow-up, 12 and 71% of trial participants were expected to have been re-infected when the respective EIRs were 1 and 10. By 730 days of follow-up, 63 and 100% of trial participants were now expected to have been re-infected at respective EIRs of 1 and 10.

A supplementary analysis using a simple, hazard-based model confirmed the effect of the mean time to first relapse on efficacy estimates (Additional file 1: Fig. S13). A diminished effect of the duration of follow-up was observed, though this simpler model assumed complete observation of recurrent infections and ignored the effects of anti-parasite and clinical immunity. This underscores the importance of using an individual-based model that accommodates specific and detailed descriptions of the trial design and transmission process in order to predict biases that are likely to occur in an actual trial context.

### Effect of vector control

Higher transmission intensity biased efficacy estimates downward, because trial participants in both the treatment and control arms were frequently re-infected (Fig. 2A). This analysis assessed the potential of LLINs and IRS to protect trial participants from reinfection and therefore reduce the downward bias due to transmission intensity. Because the effects of LLINs and IRS depend upon vector bionomics, the absolute proportion of bites that occurred indoors and the absolute proportion of bites that occurred while trial participants were in bed were varied.

**Fig. 5:**
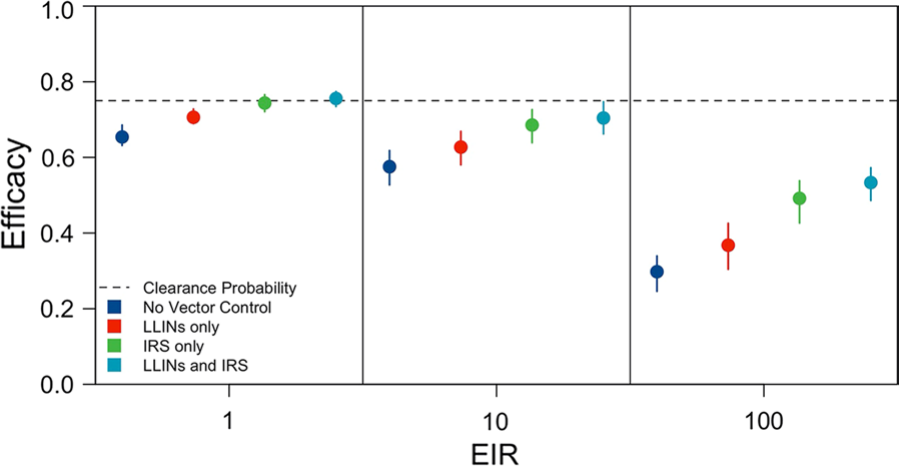
Effect of vector control on efficacy estimates. The impact of LLIN distribution (red), IRS administration (green), and combined LLIN distribution and IRS administration (teal) on LM-detectable recurrence-free efficacy estimates is compared to a no-intervention scenario (dark blue) across a range of EIRs. Each point represents the median of 200 simulations, and each bar is the interquartile range. The dotted line is the clearance probability simulated in each trial. The absolute proportion of bites occurring indoors (${\Phi }_{\mathrm{I}}$) was 0.90 and in bed (${\Phi }_{\mathrm{B}}$) was 0.45

Vector control interventions were effective in reducing the downward bias due to transmission intensity in trial locations where the vector was predominantly endophagic (i.e., feeds indoors) (Fig. 5). With an endophagic vector with no preference in biting time and at an EIR of 10, the estimated efficacies improved from 0.58 (0.53–0.62) in the absence of vector control to 0.63 (0.58–0.67) with LLINs, 0.69 (0.64–0.73) with IRS, and 0.70 (0.66–0.75) with the combination of LLINs and IRS. By comparison, with an exophagic (i.e., feeds outdoors) vector with no preference in biting time and at an EIR of 10, efficacy estimates improved only slightly from 0.57 (0.52–0.61) in the absence of vector control, 0.57 (0.52–0.61) with LLINs, and 0.57 (0.52–0.61) with IRS to 0.58 (0.55–0.63) with the combination of LLINs and IRS (Additional file 1: Fig. S3D).

Across all mosquito biting behaviours simulated, the combination of LLINs and IRS was generally more effective than either intervention in isolation in reducing the downward bias in efficacy estimates (Additional file 1: Fig. S3). When considering interventions in isolation, IRS was more effective than LLINs in reducing the downward bias, and the distribution of LLINs most improved efficacy estimates under scenarios in which an appreciable number of mosquito bites were taken at night while trial participants were in bed.

### Effect of parasite genotyping

Unlike vector control interventions that reduce the downward bias in efficacy by reducing the number of re-infection events among trial participants (Additional file 1: Fig. S4), parasite genotyping used in combination with time-to-event data could correct or reduce the downward bias by distinguishing recurrent infection events that directly reflect the clearance probability (i.e., relapses arising from hypnozoite broods acquired prior to treatment) from all other recurrent infections. Simulations assessed the potential of a generic method leveraging genotyping and time-to-event data to correct the bias in efficacy estimates at different transmission intensities and considered how performance characteristics of this method affected the extent to which the bias was corrected by varying its sensitivity and specificity. For the purpose of this simulation study, sensitivity is defined as the probability of correctly identifying relapses associated with hypnozoite broods acquired prior to treatment, and specificity is defined as the probability of correctly identifying other recurrent infections (i.e., reinfections and relapses associated with hypnozoite broods acquired after treatment).

**Fig. 6:**
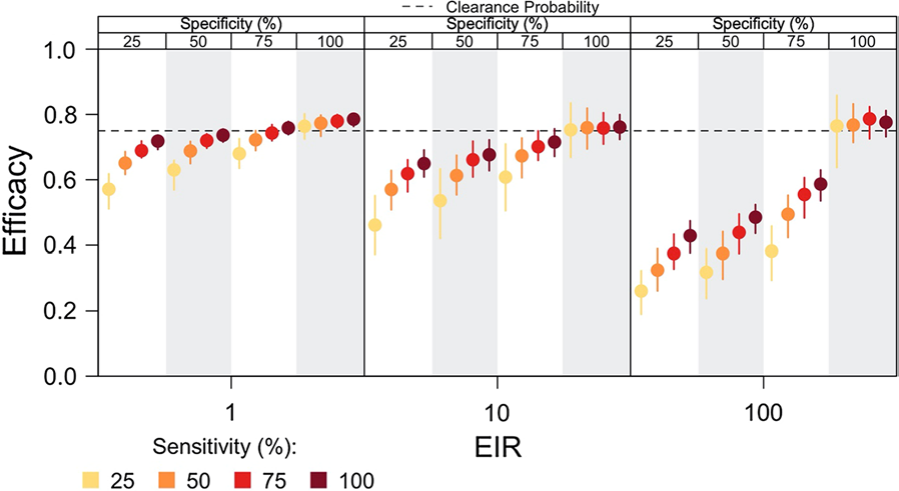
Effect of parasite genotyping on efficacy estimates. The impact of genotyping recurrent infections to estimate the efficacy of radical cure is shown for different sensitivities and specificities of the genotyping method across a range of EIRs. Sensitivity of the genotyping method is the probability of correctly identifying a relapse associated with hypnozoites acquired prior to treatment, and specificity of the genotyping method is the probability of correctly identifying other recurrent infections. For a given EIR, the vertical panels correspond to the specificity of the genotyping method, and the colours of the points within a given panel denote the sensitivity of the genotyping method. Each point represents the median of 200 simulations, and each bar is the interquartile range. The dotted line is the clearance probability simulated in each trial

At lower and intermediate transmission intensities (i.e., EIRs of 1 and 10), efficacy estimates changed more with improved sensitivity than improved specificity of the method (Fig. 6). By contrast, at higher transmission intensities, high specificity was needed to reduce the downward bias in efficacy. At an EIR of 100 and assuming 100% sensitivity of the method, the estimated efficacy of an 8-aminoquinoline with clearance probability equal to 0.75 improved from 0.43 (0.38–0.47) at 25% specificity to 0.78 (0.73–0.81) at 100% specificity. Under the alternative scenario in which specificity was 100%, the estimated efficacy at the respective EIR did not significantly change with improved sensitivity and was 0.76 (0.64–0.86) at 25% sensitivity and 0.78 (0.73–0.81) at 100% sensitivity. The specificity was more important at higher EIR, because at an EIR of 100, 100% of participants were expected to be re-infected during follow-up (Fig. 4C). Misclassifying re-infection events as failures of 8-aminoquinoline treatment (i.e., relapses associated with hypnozoite broods acquired prior to treatment) led to a greater downward bias, so a highly specific method was needed in settings where trial participants were frequently re-infected. These effects were less pronounced at low transmission intensities, given that fewer trial participants were re-infected during follow-up.

### Effect of the 8-aminoquinoline

The previous analyses considered trials of an 8-aminoquinoline that provided prophylaxis for 28 days, a duration of less than the 32-day period of left-censoring used to calculate efficacy. Consequently, prophylaxis did not impact efficacy estimates. To examine whether a longer duration of prophylaxis biased efficacy estimates, trials were simulated for two different 8-aminoquinolines, PQ and TFQ, that provide prophylactic effects for fixed periods of 28 and 45 days, respectively. Because the benefit of prophylaxis increases with transmission intensity, trials were simulated at EIRs equal to 1, 10, and 100, assuming homogeneous biting.

**Fig. 7:**
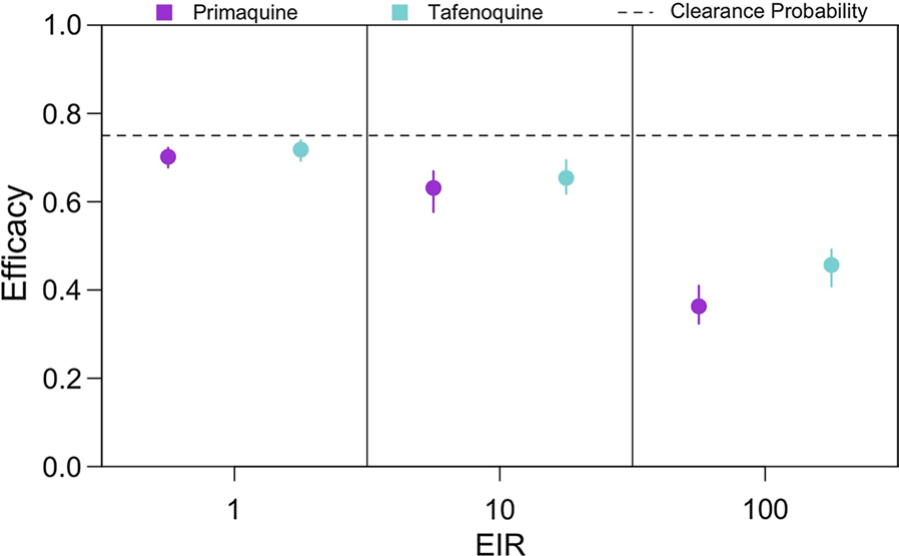
Effect of 8-aminoquinoline on efficacy estimates. LM-detectable recurrence-free efficacy estimated from simulated clinical trials is shown for PQ (purple) and TFQ (blue) at different EIRs. Each point is the median of 200 simulations, and each bar is the interquartile range. The dotted line is the clearance probability simulated in each trial

A longer duration of prophylaxis biased our efficacy estimates upward, though the magnitude of the bias depended upon transmission intensity (Fig. 7). In a low-transmission setting (i.e., EIR of 1), efficacy estimates did not change much with an increased duration of prophylaxis, because a median of 1.3% (1.1–1.6%) of participants in the control arm were re-infected between days 32 and 45 post-enrolment, the time period over which TFQ provided prophylactic effects not accounted for by left-censoring. Consequently, at an EIR of 1, the estimated efficacies of TFQ and PQ were 0.72 (0.69–0.74) and 0.70 (0.68–0.72), respectively. At higher EIRs, there was a greater estimated efficacy for TFQ than PQ, because participants in the control arm were frequently reinfected whereas participants in the treatment arm were protected from reinfection. On average, the percentage of participants in the control arm who were re-infected between days 32 and 45 post-enrolment was 10% (9.5–11%) and 54% (52–55%) at EIRs of 10 and 100, respectively. Consequently, the respective estimated efficacies of TFQ and PQ were 0.65 (0.62–0.69) and 0.63 (0.58–0.67) at an EIR of 10 and 0.46 (0.41–0.49) and 0.36 (0.32–0.41) at an EIR of 100.

### Effect of the efficacy metric and infection endpoint

To test whether the efficacy estimates obtained were sensitive to the choice of efficacy metric and the infection endpoint, clinical trials were simulated in which trial participants were assessed for PCR-detectable, LM-detectable or clinical infections during follow-up. Trials were simulated at EIRs of 1, 10 and 100 assuming homogeneous biting, and efficacy was calculated using three metrics: (1) Cox proportional hazards model; (2) incidence rates; and, (3) the proportion at risk.

**Fig. 8:**
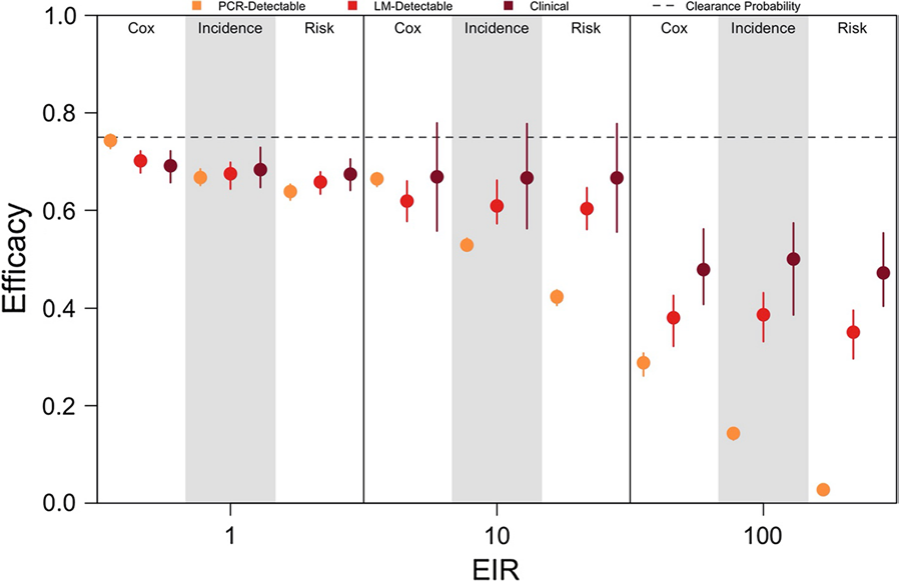
Effect of efficacy metric and infection endpoint on efficacy estimates. Efficacy estimates obtained from simulated clinical trials at different EIRs is shown when calculated using the Cox proportional hazards model, incidence rates or the proportion at risk. The infection endpoint was clinical (maroon), LM-detectable (red), or PCR-detectable (orange) recurrent infections identified during follow-up. Each point is the median of 200 simulations, and each bar is the interquartile range. The dotted line is the clearance probability simulated in each trial

For all three efficacy metrics considered, increasing transmission intensity caused a downward bias in efficacy estimates, because at higher transmission intensities, most participants in both the treatment and control arms experienced a re-infection or relapse during follow-up (Fig. 8). For PCR-detectable infections, estimated efficacy was highest if calculated using the Cox proportional hazards model and lowest if calculated using the proportion at risk. This suggested that, in the absence of parasite genotyping, the magnitude of bias caused by re-infection from mosquito biting was greater when basing efficacy upon incidence rates or the proportion at risk than upon proportional hazards.

The sensitivity of the assay by which trial participants were assessed for recurrent infections also affected efficacy estimates, though the direction of the effect depended upon the chosen efficacy metric. For efficacy estimates based upon incidence rates or the proportion at risk, shifting from a more sensitive assay (i.e., PCR-based detection) to a less sensitive assay (i.e., monitoring for clinical infections) reduced the downward bias due to re-infection, particularly at higher transmission intensities. This was due to the fact that the fraction of recurrent infections ascertained increased with a more sensitive assay, causing us to detect more recurrent infections during follow-up and making the incidence rates and proportions at risk appear more similar across the treatment and control arms. By contrast, using the Cox proportional hazards model, the relationship between efficacy estimates and the assay sensitivity depended upon transmission intensity. Across all transmission intensities, clinical infections both occurred less frequently and were detected later than PCR- and LM-detectable infections. Therefore, at an EIR of 1 where re-infection from mosquito biting was less frequent, a more sensitive assay more accurately captured differences in the timing of recurrent infections across trial arms that were attributable to the effect of radical cure. By contrast, at an EIR of 100, nearly all trial participants were re-infected during follow-up. At this transmission intensity, a more sensitive assay instead made the timing of recurrent infections appear more similar across trial arms. Thus, using a less sensitive assay improved efficacy estimates. The non-monotonic relationship observed at the intermediate transmission intensity (i.e., EIR of 10) reflects a change between these two extremes.

## Discussion

Obtaining standardized estimates of the effect of radical cure on *P. vivax* hypnozoites is challenging, because the recurrent infection endpoint used in clinical trials includes infection events, such as re-infections by mosquito biting, that do not reflect the effect of radical cure on *P. vivax* hypnozoites. This simulation study identified features of the trial setting, including transmission intensity, heterogeneous feeding patterns, and the relapse rate of the *P. vivax* parasite, that affected estimates of drug efficacy and the utility of clinical trial data to assess the extent to which radical cure prevents relapse (Table 2). It demonstrated that the use of vector control and genotyping methods are two approaches that can reduce and, in some cases, even correct these site-specific biases and yield more standardized estimates of drug efficacy against relapse.

**Table 2:** Summary of identified biases

Feature of trial setting	Direction of bias	Cause of bias
Transmission intensity (EIR)	Downwards	At higher transmission intensities, there is more frequent reinfection and reduced detectability of recurrent infections
Relapse rate	Downwards	With a longer time to first relapse, fewer trial participants relapse during follow-up
Duration of follow-up	Downwards	With a longer duration of follow-up, more trial participants have an observed reinfection by mosquito biting
Duration of prophylaxis	Upwards	With a longer duration of prophylaxis, fewer trial participants are reinfected by mosquito biting
Efficacy metric	Downwards	Measured efficacy is lower when based upon incidence rates or proportion at risk than upon proportional hazards
Assay sensitivity	Downwards	For incidence rates and the proportion at risk, a more sensitive assay detects more recurrent infections
Intervention action	Upwards	Efficacy based upon proportional hazards is biased upwards for an “all-or-none” intervention, because the “all” group increases the person-time at risk

A complete description, including the direction and cause, of the biases identified in this analysis are provided

In the recent GATHER, DETECTIVE and IMPROV trials [7–10], efficacy estimates varied across trial sites that spanned different transmission intensities and relapse phenotypes. Interpreting site-specific differences in efficacy is important for past and future clinical trials [35], and these simulation results suggest that the differences in efficacy estimates could be caused by site-specific biases that arise when the infection endpoint used to measure efficacy does not directly reflect the action of the therapeutic being trialed. If the trial sites vary as regards transmission intensity, these results suggest that efficacy estimates could be lower in high-transmission settings, because trial participants are more frequently re-infected during follow-up, and the detectability of their recurrent infections is reduced. Greater heterogeneity in mosquito biting reduces this downward bias by reducing the number of trial participants who are bitten and possibly re-infected during follow-up, though this effect is non-monotonic at very high transmission intensities due to the predicted interaction with anti-parasite immunity. Trial sites may also differ in the rates at which *P. vivax* hypnozoites activate [19]. A slower rate of relapse (i.e., a longer time to relapse) implies that an appreciable number of trial participants will not yet have relapsed during follow-up, thereby biasing efficacy estimates downward [36]. Increasing the duration of follow-up further compounds the bias rather than corrects it, because a long duration of follow-up causes more participants in both the treatment and control arms to have an observed re-infection by mosquito biting, particularly at higher transmission intensities. Finally, frequent re-infection at higher transmission intensities makes the comparison of treatment regimens with different durations of prophylaxis challenging, because a longer duration of prophylaxis prevents more individuals in the treatment arm from becoming re-infected. Ignoring differences in prophylaxis may lead to an overestimate of the effect against hypnozoites for treatment regimens that provide a longer period of prophylaxis, such as TFQ co-administered with CQ, relative to other treatment regimens that provide a shorter period of prophylaxis, such as PQ co-administered with CQ.

In general, there was greater bias in high-transmission settings, because of the higher risk of recurrent infections being due to re-infections from new mosquito bites. In low-transmission settings, this bias is reduced, although there is the concomitant challenge of recruiting sufficient trial participants. This could be addressed by extending the length of the trial, though this may be financially and logistically impractical, by recruiting travellers to high-transmission settings, or by conducting controlled human malaria infections. These simulation results suggest that trial investigators can reduce bias by prospectively preventing reinfections with vector control or by retrospectively accounting for re-infections by leveraging time-to-event and parasite genotyping data.

LLINs and IRS were predicted to be most effective in trial settings with an endophagic vector that bites mostly indoors, so novel vector control interventions, such as spatial repellents, may be needed in settings with an exophagic vector to reduce peri-domestic biting [37], particularly in the early evening. Distribution of LLINs occurred as part of the DETECTIVE trial [7–9], suggesting the feasibility of implementing vector control in a trial context. Entomological data collected prior to trial enrolment could characterize the local vector bionomics and inform the selection of interventions [38, 39]. Moreover, blood meal analysis [40–42] or serological assays [43, 44] performed as part of the trial could quantify heterogeneous feeding, a factor that directly affects the magnitude of the site-specific bias.

These results demonstrate that parasite genotyping could be used in concert with time-to-event data to generate less biased efficacy estimates that better reflect the effect of the 8-aminoquinoline against hypnozoite broods. Although in practice the sensitivity of a genotyping method may be reduced by not observing infections that occurred prior to trial enrolment, the extent to which bias was reduced in the analysis was robust to changes in sensitivity of the method and depended most upon its specificity. High specificity can likely be achieved given the high expected heterozygosity of microsatellite and amplicon deep sequencing panels, as well as the ability to detect minority clones in multiclonal *P. vivax* infections [45–49]. Parasites sampled during follow-up were genotyped in the DETECTIVE and IMPROV trials, though the genotyped samples were not used to correct efficacy estimates due to the challenges inherent in doing so [7–10]. Statistical frameworks leveraging genotyping and time-to-event data and accounting for genetic relationships among parasites to distinguish relapses from re-infections have been successfully developed and could be readily integrated into the analysis of past and future clinical trial data [14, 49]. Nevertheless, this approach may be insufficient to overcome biases introduced by improper trial design, such as a short duration of follow-up relative to the relapse rate in the trial location. Furthermore, higher performance characteristics may be unachievable at higher transmission intensities, where the detectability of recurrent infections is reduced and higher hypnozoite burdens and frequent re-infections limit the information content of genotyping data.

Beyond the factors that make resolving differences in efficacy challenging within the context of a single trial, this simulation study identified features of trial design that could limit the comparability of efficacy estimates across trials. Specifically, the details of how the efficacy endpoint was calculated (i.e., time to first infection *vs* incidence ratio) and the sensitivity of the assay used to detect recurrent infections resulted in considerable variation in efficacy estimates, particularly at higher transmission intensities. These results suggest that future studies performing meta-analyses of the efficacy of radical cure should consider differences in the trial designs of the clinical trials included.

There are a number of limitations of this analysis. First, the model was not calibrated to clinical trial data, so the results are not representative of any specific trial settings. However, the simulations encompassed a wide range of epidemiological settings, so the site-specific biases identified in this analysis should reflect variation possible across trial sites. Nevertheless, future work could directly quantify the magnitude of these site-specific biases by fitting transmission models directly to clinical trial data collected from trial sites that vary with respect to transmission intensity, heterogeneity in biting, and relapse rate. Second, there remains much about *P. vivax* biology that is not well understood [50, 51], so the simplified representation of hypnozoite activation and death in the transmission model may not fully capture reality. Third, it did not account for the effect of cytochrome P-450 polymorphisms on drug efficacy directly. Although little difference was observed in efficacy estimates under the all-or-none and leaky responses when based upon Cox proportional hazards, future work could examine this potential source of variance in greater detail. Finally, there remains an imperfect understanding of the acquisition and loss of anti-parasite and clinical immunity [52]. Underestimating the level of anti-parasite and clinical immunity among trial participants may lead to a greater fraction of recurrent infections being detected during follow-up than would occur in an actual trial context. This could have led to an overestimation of site-specific biases, in which case more refined mathematical representations of immunity could be beneficial for future studies building on this analysis [11, 33].

## Conclusions

This analysis predicts that site-specific biases are likely to occur in clinical trials for radical cure, and these results suggest that care should be taken in the planning of future trials and the interpretation of trial data. Mathematical modelling that accounts for site-specific biases can aid in the interpretation of clinical trial data and may be useful for identifying future trial sites where biases may be less severe [53]. As always, the utility of the insights from mathematical models is improved with additional data, so modelling should be integrated into clinical trial design to identify data needs and inform data collection in order to reduce these biases and improve understanding of radical cure’s potential to control *P. vivax* malaria.

## Supplementary Information

**Additional file 1**: **S1** Text. Supplementary method and results.

## Data Availability

The datasets generated during and/or analysed during the current study are available in the GitHub repository, https://github.com/johnhhuber/Radical_Cure_Uncertainty.

## Abbreviations

CQ: Chloroquine
PQ: Primaquine
TFQ: Tafenoquine
CYP2D6: Cytochrome P-450 isoenzyme 2D6
EIR: Entomological inoculation rate
LM: Light microscopy
PCR: Polymerase chain reaction
G6PD: Glucose-6-phosphate dehydrogenase
LLIN: Long-lasting insecticidal net
IRS: Indoor residual spraying
